# Diet Overlap and Foraging Activity between Feral Pigs and Native Peccaries in the Pantanal

**DOI:** 10.1371/journal.pone.0141459

**Published:** 2015-11-04

**Authors:** Mauro Galetti, Hiléia Camargo, Tadeu Siqueira, Alexine Keuroghlian, Camila I. Donatti, Maria Luisa S. P. Jorge, Felipe Pedrosa, Claudia Z. Kanda, Milton C. Ribeiro

**Affiliations:** 1 Departamento de Ecologia, Universidade Estadual Paulista (UNESP), Rio Claro, São Paulo, Brazil; 2 Wildlife Conservation Society, Campo Grande, Mato Grosso do Sul, Brazil; 3 The Betty and Gordon Moore Center for Science and Oceans, Conservation International, Arlington, Virginia, United States of America; 4 Depatment of Earth & Environmental Sciences, Vanderbilt University, Nashville, Tennessee, United States of America; 5 Programa de Pós-graduação em Ecologia e Biodiversidade, Universidade Estadual Paulista (UNESP), Rio Claro, São Paulo, Brazil; University of Sassari, ITALY

## Abstract

Inter-specific competition is considered one of the main selective pressures affecting species distribution and coexistence. Different species vary in the way they forage in order to minimize encounters with their competitors and with their predators. However, it is still poorly known whether and how native species change their foraging behavior in the presence of exotic species, particularly in South America. Here we compare diet overlap of fruits and foraging activity period of two sympatric native ungulates (the white-lipped peccary, *Tayassu pecari*, and the collared peccary, *Pecari tajacu*) with the invasive feral pig (*Sus scrofa*) in the Brazilian Pantanal. We found high diet overlap between white-lipped peccaries and feral pigs, but low overlap between collared peccaries and feral pigs. Furthermore, we found that feral pigs may influence the foraging period of both native peccaries, but in different ways. In the absence of feral pigs, collared peccary activity peaks in the early evening, possibly allowing them to avoid white-lipped peccary activity peaks, which occur in the morning. In the presence of feral pigs, collared peccaries forage mostly in early morning, while white-lipped peccaries forage throughout the day. Our results indicate that collared peccaries may avoid foraging at the same time as white-lipped peccaries. However, they forage during the same periods as feral pigs, with whom they have lower diet overlap. Our study highlights how an exotic species may alter interactions between native species by interfering in their foraging periods.

## Introduction

Inter-specific competition is considered one of the main selective pressures affecting species distribution and coexistence [1]. Different species vary their foraging strategies in order to minimize encounters with conspecifics [2] and predators [3]. Foraging activity includes partitioning of diet and time, and is recognized as an important factor for species maintenance; however, it is still poorly known whether and how native species change their foraging behaviour in the presence of exotic species in tropical ecosystems (but see [4]).

Feral pigs (*Sus scrofa*) have been introduced in many parts of the world [5, 6] and because they are generalists in terms of diet and habitat use, they can potentially compete with native species [7]. In South America, it is thought that feral pigs affect three co-occurring native peccaries, i.e., white-lipped peccary (*Tayassu pecari*), collared peccary (*Pecari tajacu*) and Chacoan peccary (*Catagonus wagneri*), which occupy similar trophic levels [8–10]. In the Pantanal, one of the largest floodplains in the world the two native peccaries (collared and white-lipped peccaries) and the exotic feral pigs, have co-existed for over 200 years [10]. Several authors have suggested weak competition between peccaries and feral pigs [10, 11], although the dominance of fruits (a patchy and ephemeral resource) in peccaries and feral pigs diets is well recognized [10, 12, 13]. The inference of co-occurrence effects between feral pigs and the two peccaries is based mainly on habitat use and fecal analyses [10]. Nonetheless, the presence of the larger feral pig in a region with a dramatic fruit scarcity period during the dry season may change the foraging behavior of the native species [12].

**Fig 1:**
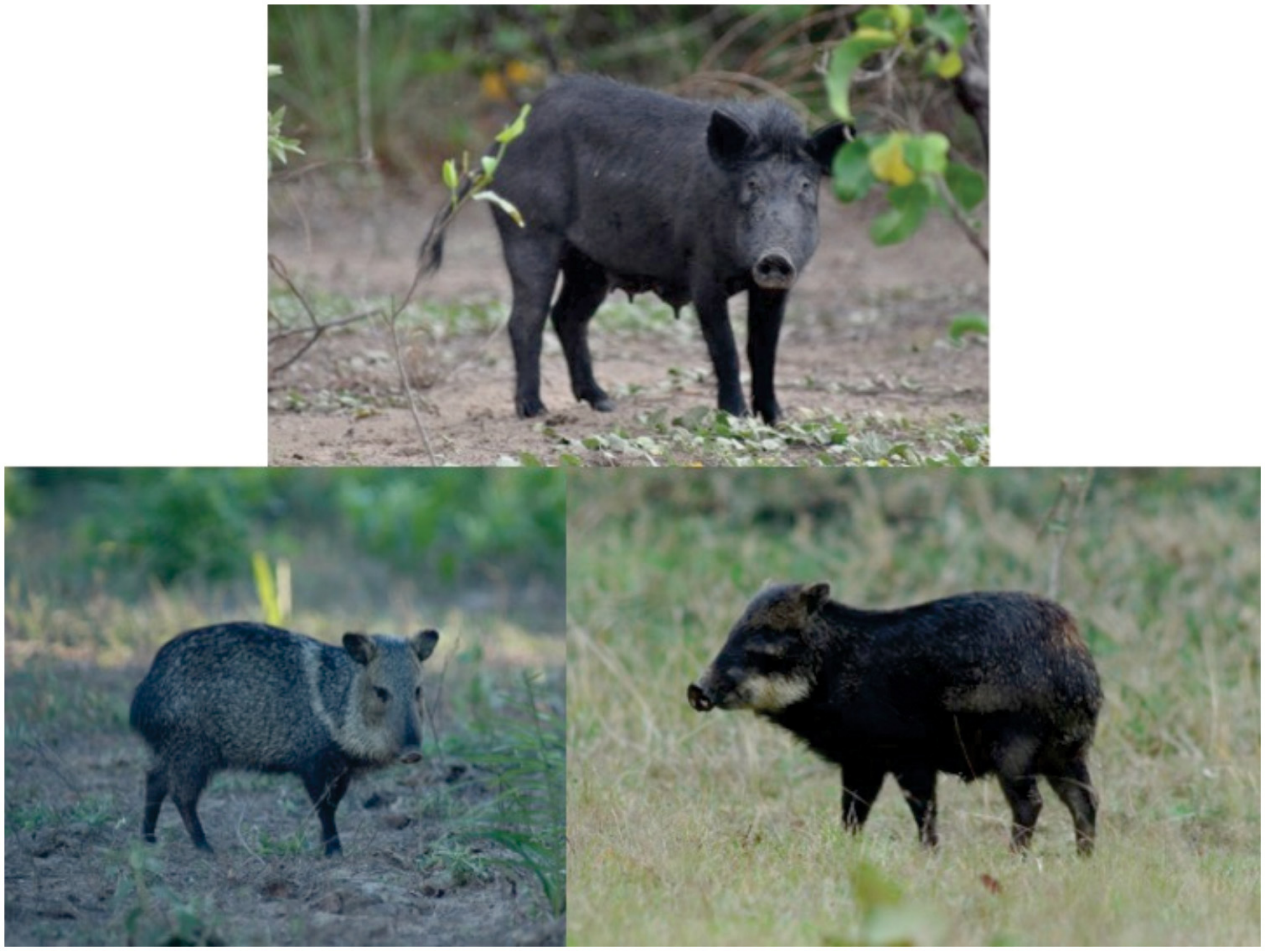
Feral pigs (*Sus scrofa*) (Upper center), collared peccaries (*Pecari tajacu*) (Botton left) and white-lipped peccaries (*Tayassu pecari*) (Botton right) in the Brazilian Pantanal.

Here, we used long-term camera-trap sampling placed underneath 37 fruiting species to investigate the diet overlap and foraging activity period of two sympatric peccaries (*Tayassu pecari* and *Pecari tajacu*) and exotic feral pigs (*Sus scrofa*) (Fig 1) in the Brazilian Pantanal. We were particularly interested in testing whether: (1) exotic and native species share the same fruiting resources, and (2) if feral pigs can affect the period of native peccary foraging activities.

## Materials and Methods

### Study area

The Pantanal is one of the largest floodplains in the world (14° to 22°S and 53° to 66°W) with an average annual rainfall of 1,100 mm, and a wet season from November to March. The Pantanal landscape is a complex mosaic of tropical forest, savanna, and aquatic environments adapted to a highly variable annual and multi-annual flood cycles that can last as long as six months and covers as much as 110,000 km^2^ [14, 15]. The region has a high diversity of habitats and the vegetation is composed of isolated clumps of trees (*capões*), non-floodable savannas (*cordilheiras*), grasslands (*campos*), riparian forests, and a large variety of lakes (*baias*), with high abundance of aquatic plants, and soda lakes (*salinas*). The Pantanal supports a highly productive and diverse assemblage of neotropical flora and fauna [16]. Cattle and other livestock were introduced by European colonists to the Pantanal in the mid-1500s [17].

**Fig 2:**
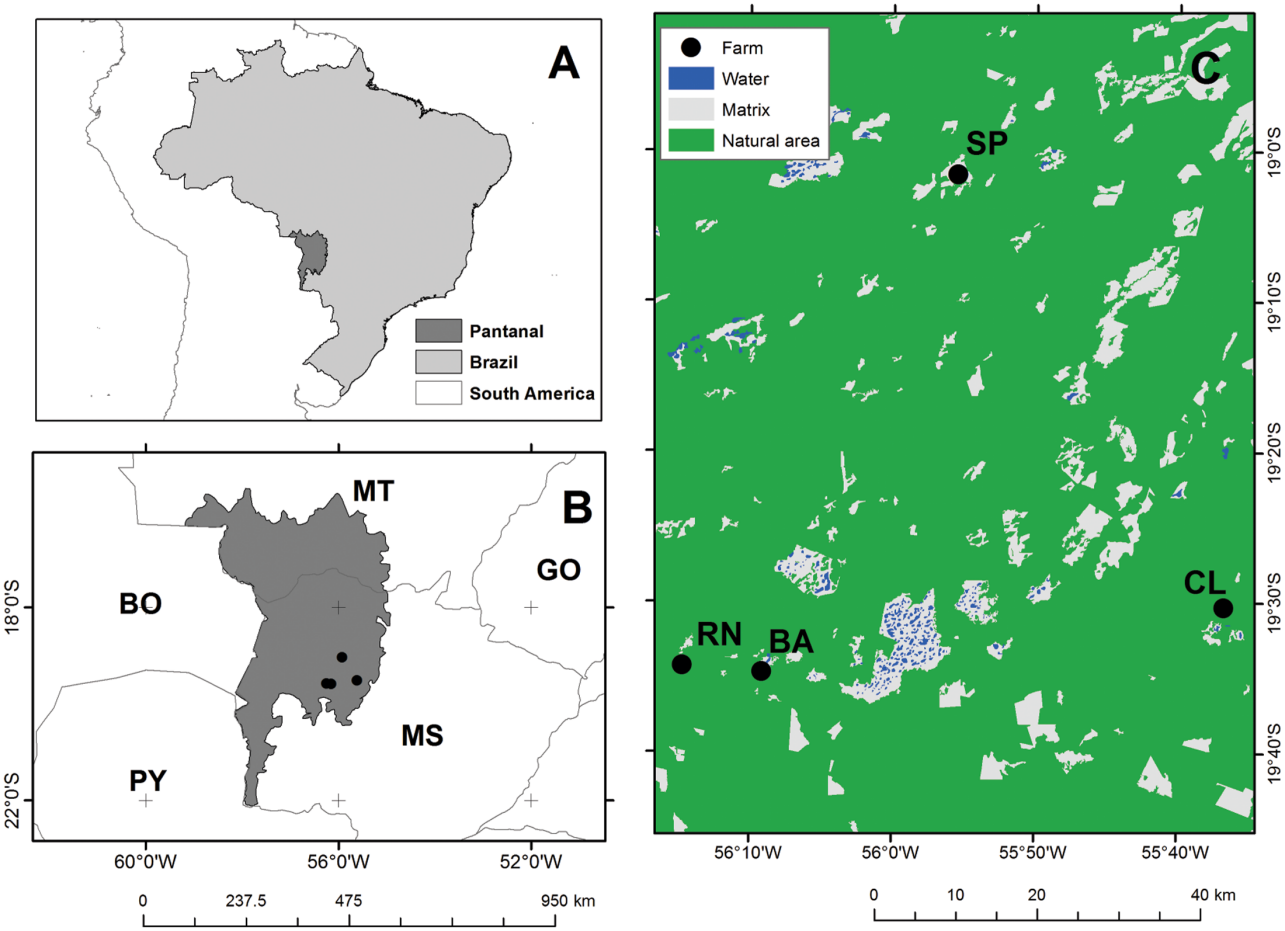
Location of the Brazilian Pantanal and the study sites. Fazenda Rio Negro (RN), Fazenda Barranco Alto (BA), Campo Lourdes (CL) and São Paulino (SP).

We collected data at five different farms: Rio Negro, Barranco Alto, São Paulino, Campo Lourdes and Santa Emilia, all located in Nhecolândia, one of the largest sub regions of the southern Pantanal (Fig 2). All areas have peccaries and feral pigs. Rio Negro Farm (19°34’15”S and 56°14’43”W) is a 7,700-ha privately owned ranch in which 80% was set aside as a protected private reserve (RPPN). Barranco Alto Farm (19°34’40”S and 56°09’08”W) is an 11,000-ha privately owned ranch, of which 45% is protected since 1980. Both areas are among the most well-preserved areas in the lower Rio Negro region of the Pantanal. São Paulino Farm (19°01’33”S and 55°55’31”W) is an 11,000-ha privately owned ranch located between the Taquari and Negro rivers. Campo Lourdes and Santa Emilia farms (19°30’18” S and 55°36’44”W) are private owned ranches and together occupy 8,300 ha.

The average group size of feral pigs is 8.6 animals and they reach a density 6.35 individual/km^2^. White-lipped peccaries forage in large herds (up to 100 animals) reaching a density of 9.63 individual/km^2^ and collared peccaries forage in small herds (mean of 4 animals) with a density 3.69 individual/km^2^ (Galetti et al. unpublished data).

In the lower Rio Negro ecoregion of the southern Pantanal, weight of adult male feral pigs is reported as 60.6 kg (n = 17, SD = 17.4) and females weighed 42.4 kg (n = 15, SD = 15.0) (A. Desbiez and A. Keuroghlian, unpublished data). Both native peccaries are smaller than feral pigs: white-lipped peccaries in the same region weight 32.3 kg (SD = 3.64) [18] and collared peccaries 17.6 kg (n = 21, SD = 2.0) [19]. Collared peccary’s home range can reach a maximum of 305 ha [20], while white-lipped peccary’s varies between 7,585 and 8,037 ha [19] and feral between 1,432 and 610 ha, (A. Desbiez and A. Keuroghlian, unpublished data).

### Diet Overlap and Foraging Activity Period

Between 2003 and 2010 we placed infra-red camera traps beneath 37 fruiting species in order to sample diet overlap and frugivore activity of terrestrial mammals [21]. We organized all images in a database in which frugivores were identified and their activity period was recorded (See S1 Table). As not all photos captured the animals eating the fruits, we validated our data with information collected through fecal analyses and focal observations [21, 22]. For the diet-overlap analysis we considered a “feeding bout” when a frugivore species was recorded foraging at a fruiting tree [23]. For the foraging-activity-period analysis we defined consecutive photos of the same species as independent occurrences if the interval between photos was greater than 30 min. Data were grouped in one-hour intervals, from 0:00 to 24:00 hours [24]. To discriminate peccaries in the presence from peccaries in the absence of feral pigs, we considered each fruiting tree as a sample unit in the foraging-activity-period analysis. During the period of sampling of a fruiting tree (which was, in general, the hole fruiting period), if no feral pig was recorded it meant absence of feral pigs. Then, we compared the frequency of photos of *T*. *pecari* and *P*. *tajacu* in a combination of presence and absence of feral pigs.

We used Pianka’s index of niche overlap, which varies from 0 (complete dietary separation) to 1 (complete overlap), to estimate diet overlap among all unique pairs of species (*Pecari tajacu*, *Tayassu pecari* and *Sus scrofa*) [25]. We compared observed values of the index with values generated by null model analysis. Following Gotelli and Graves [26], we used the randomization algorithm 3 [27] to generate the null expectation. This algorithm keeps the observed niche breadth of each species, but shuffles resource categories. Observed values were considered higher than would be expected by chance if *P* < 0.05.

We computed circular statistics to determine overall timing of species activity and circular analysis of variance in R (function ‘‘circular aov”, package “circular”, [28]) to investigate variation in the timing of activity (time that the photo was taken) of each species. To investigate the effect of *Sus scrofa* on the activity time of the two native peccaries, we compared activity time at the fruiting tree level (fruiting trees where they co-occurred and did not co-occur with *Sus scrofa*) among the three species, since *Pecari tajacu*, *Tayassu pecari* and *Sus scrofa* co-occurred in all study areas.

## Results

### Diet overlap

**Fig 3:**
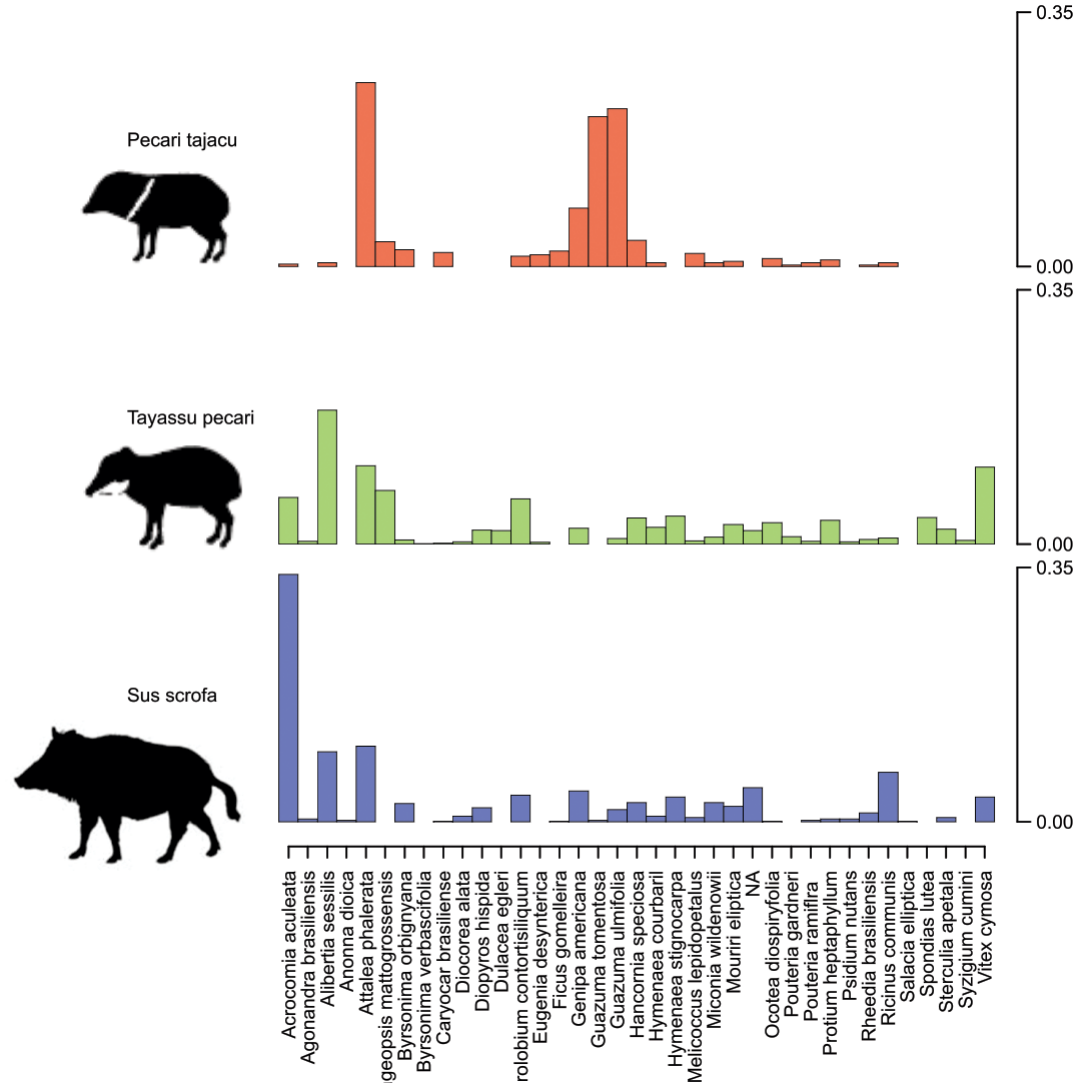
Diet overlap of fruits among white lipped and collared peccaries (*Pecari tajacu*, *Tayassu pecarie*), and feral pigs (*Sus scrofa*) in the Brazilian Pantanal. Each bar represents the proportion of independent photos taken for each plant species.

The sampling effort reached over 1,637 camera days, totalling 3,096 “feeding bout”, including 558 photos of *Pecari tajacu*, 1,510 of *Tayassu pecari* and 1,028 of *Sus scrofa* in 37 fruiting trees in the Pantanal (Fig 3 and Table 1). Since fruiting trees differ in abundance (some common and others rare) and in amount of fruits produced annually, camera day effort could not be equally distributed among all 37 species.

**Table 1:** Sampling effort and number of photos taken in fruiting trees visited by natives white-lipped peccaries (*Tayassu pecari*) and collared peccaries (*Pecari tajacu*) and by an exotic feral pigs (*Sus scrofa*) in the Pantanal, Mato Grosso do Sul, Brazil.

Plant Species	Camera.day effort	*Pecari tajacu*	*Tayassu pecari*	*Sus scrofa*
*Acrocomia aculeata*	72.25	2	97	350
*Attalea phalerata*	253.14	141	163	107
*Alibertia sessilis*	70.28	3	278	99
*Metrodorea nigris*	32.42	3	13	70
Not Identified	42.49	0	28	48
*Genipa americana*	184.81	45	33	44
*Enterolobium contortisiliquum*	42.66	8	94	38
*Hymenaea stignocarpa*	20.42	0	58	35
*Vitex cymosa*	34.26	0	160	35
*Hancornia speciosa*	134.15	20	54	27
*Miconia wildenowii*	31.81	3	15	27
*Byrsonima orbignyana*	48.38	13	9	26
*Mouriri eliptica*	69.28	4	41	22
*Diopyros hispida*	12.38	0	29	20
*Guazuma ulmifolia*	153.89	121	12	17
*Rheedia brasiliensis*	53.73	1	10	13
*Diocorea alata*	8.52	0	5	8
*Hymenaea courbaril*	31.38	3	35	8
*Melicoccus lepidopetalus*	15.87	10	7	6
*Sterculia apetala*	44.37	0	31	6
*Agonandra brasiliensis*	8.14	0	6	4
*Protium heptaphyllum*	29.28	5	50	4
*Psidium nutans*	22.79	0	5	4
*Anonna dioica*	26.99	0	0	2
*Guazuma tomentosa*	21.92	115	0	2
*Pouteria ramiflora*	2.14	3	6	2
*Caryocar brasiliense*	22.04	11	2	1
*Ficus gomelleira*	28.61	12	0	1
*Ocotea diospiryfolia*	21.96	6	45	1
*Salacia elliptica*	11.94	0	0	1
*Bocageopsis mattogrossensis*	23	19	112	0
*Byrsonima verbascifolia*	7.88	0	1	0
*Dulacea egleri*	25.28	0	28	0
*Eugenia desynterica*	11.89	9	4	0
*Pouteria gardneri*	7.02	1	16	0
*Spondias lutea*	2.02	0	55	0
*Syzigium cumini*	8.43	0	8	0
**Total**	1637,82	558	1510	1028

We found a high diet overlap between white-lipped peccaries and feral pigs (Pianka Index [PI] = 0.575, Standardized Effect Size [SES] = 2.783, *P* = 0.026), but not between collared peccaries and feral pigs (PI = 0.243, SES = 0.474, *P* = 0.161), nor between the two native species (PI = 0.331, SES = 0.575, *P* = 0.233). Feral pigs and white-lipped peccaries fed mostly on highly productive tree species, such as two species of common large-seeded palms (*Attalea phalerata* and *Acrocomia aculeata*) and one species of Rubiaceae (*Alibertia sessilis)* (Table 1). Collared peccaries, on the other hand, fed mostly in plants with small crops (*Guazuma tomentosa* and *Eugenia dysenterica*) or species with pronounced ‘masting’ years (*Ficus* spp.) (Table 1).

### Foraging Activity Period

**Fig 4:**
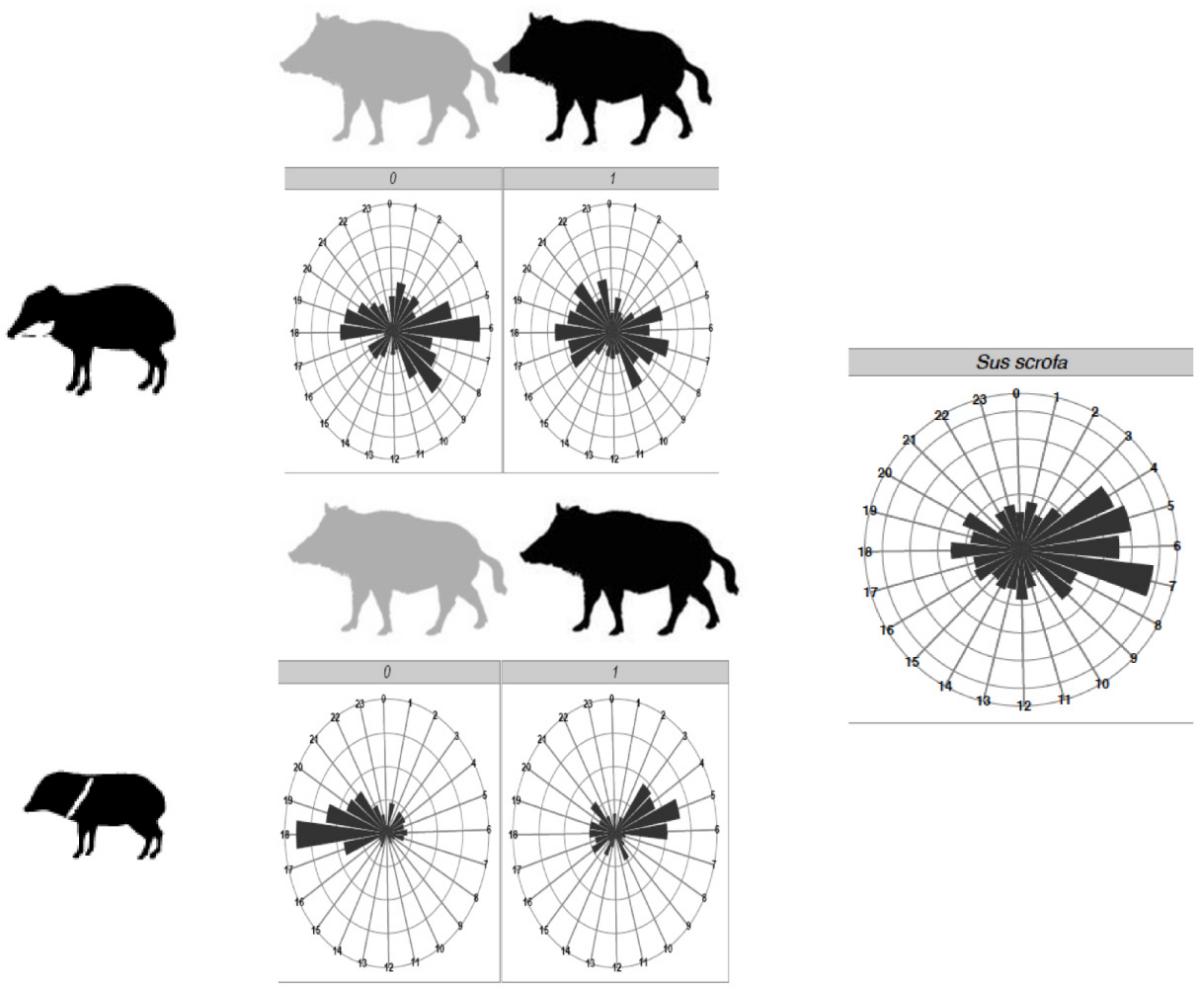
Temporal differences in the foraging activity periods under fruiting trees of native peccaries in relation to feral pigs (right) in the Brazilian Pantanal. On top: white lipped peccaries (*Tayassu pecari)* in the absence and presence of feral pigs (*Sus scrofa)*. Bottom: Collared peccaries (*Pecari tajacu)*.

We detected significant differences in feeding period between the three species (*F*_2,3093_ = 122.6, *P* < 0.001) and in the activity periods of the two peccary species in the presence or absence of foraging feral pigs. In the absence of foraging feral pigs, activity patterns of white-lipped peccaries peaked from 5:00 to 10:00 h, and that of collared peccaries peaked from 17:00 to 22:00 h. In the presence of feral pigs, white-lipped peccaries’ activity is spread throughout the day and that of collared peccaries switches to early morning (3:00 to 6:00 h) (Fig 4).

Collared peccaries showed a different activity pattern when it co-occurred with feral pigs (*F*_1,1517_ = 668, *P* < 0.001) or with white-lipped peccaries (*F*_1,1517_ = 677, *P* < 0.001)–its activity was more homogeneous throughout the day, with a small peak in the late afternoon and early evening (Fig 4). Activity patterns of *P*. *tajacu* also varied depending on the presence of *T*. *pecari* (*F*_1,547_ = 111.1, *P* < 0.001) or *S*. *scrofa* (*F*_1,547_ = 189.2, *P* < 0.001). In the absence of foraging *T*. *pecari* or *S*. *scrofa*, the collared peccary had numerous sporadic activity peaks during the day, starting at 5:00 and ending approximately at 22:00. In the presence of *T*. *pecari*, *P*. *tajacu* concentrated its activity at night and in the early morning. In the presence of the exotic *Sus scrofa*, collared peccaries were more active in the early morning (Fig 4).

## Discussion

Frugivores have developed several mechanisms to reduce direct resource competition such as changing the foraging period, habitat segregation, preference for different fruits or different parts of the same fruit or plant [22, 29]. White-lipped and collared peccaries are morphologically similar and co-occur in most of their geographic ranges, and at our study areas their diet overlap was low. It has been hypothesized that the mechanism by which white-lipped and collared peccaries partition food sources is a result of the greater masticatory force found in white-lipped peccaries, which allowed them access to nuts that collared peccaries could not crack open [8, 30]. Empirical field studies provide evidence for this hypothesis, with some fruit species preyed upon by white lipped peccaries but not by collared peccaries in the Amazon [31, 32] and in the Atlantic Forest [33]. However, the bite force differences between the two peccary species was not a clear mechanism of niche differentiation in the Pantanal [10]. Indeed, based on simultaneous radio tracking of white lipped and collared peccaries in the Atlantic Forest, showed that collared peccaries herds rapidly vacate areas when encountering white-lipped peccaries herds, suggesting that they may avoid direct competition for food with the larger white-lipped peccary. The introduction of an exotic species with a more efficient morphological system for food acquisition is expected to cause negative effects on the already existing native species [34]. Previous studies showed that feral pigs negatively affected the abundance of collared peccaries in southern Texas [34]. There is evidence that feral pigs from the Pantanal may be competing with the two native peccaries based on bite force analysis [35]. Using scat analyses and habitat use, Desbiez et al. [10] showed that diet overlap between feral pigs and the larger white-lipped was 73% and overlap between feral pigs and collared peccaries, 53%.

In our study, we observed a fine-grained spatial and temporal overlap of the three frugivorous species by identifying time of day and fruiting tree species that were visited by each species. At the dietary level, we showed that the highest degree of diet overlap was between feral pigs and white-lipped peccaries, suggesting that they can potentially compete for similar fruit resources when they are limited in the dry season [35]. Concomitantly, white-lipped peccaries showed different temporal foraging patterns in the presence of feral pigs, which may suggest that exotic pigs and white-lipped peccaries reduce the frequency of interference interactions by avoiding direct encounters. On the other hand, collared peccaries had a lower level of diet overlap with white-lipped peccaries and with feral pigs. Temporally, collared peccaries avoided white-lipped peccaries, but not feral pigs. Feral pigs may alter activity patterns of the more dominant peccary (white-lipped) allowing the less dominant peccary (collared) to switch its foraging hours to similar time periods as the introduced (and not as competitive) species. Therefore, where the three species co-occur, niche differentiation seems to be a combination of temporal separation between collared peccaries and white-lipped peccaries and between white-lipped peccaries with feral pigs, and diet separation between collared peccaries and both white-lipped peccaries and the introduced feral pig.

## Supporting Information

S1 Table Dataset of camera trapping on *Sus scrofa*, *Tayassu pecari* and *Pecari tajacu* in fruiting trees at the Brazilian Pantanal. (XLSX)
